# Desmosomes:  Essential contributors to an integrated intercellular junction network

**DOI:** 10.12688/f1000research.20942.1

**Published:** 2019-12-30

**Authors:** Kathleen J Green, Avinash Jaiganesh, Joshua A Broussard

**Affiliations:** 1 Departments of Pathology and Dermatology, Feinberg School of Medicine, Northwestern University, Chicago, IL, USA; 2 Robert H. Lurie Comprehensive Cancer Center of Northwestern University, Chicago, IL, USA

**Keywords:** Cadherins, Tight junctions, Connexins, Gap Junctions, Adherens junctions, Cytoskeleton, Intermediate Filaments

## Abstract

The development of adhesive connections between cells was critical for the evolution of multicellularity and for organizing cells into complex organs with discrete compartments. Four types of intercellular junction are present in vertebrates: desmosomes, adherens junctions, tight junctions, and gap junctions. All are essential for the development of the embryonic layers and organs as well as adult tissue homeostasis. While each junction type is defined as a distinct entity, it is now clear that they cooperate physically and functionally to create a robust and functionally diverse system. During evolution, desmosomes first appeared in vertebrates as highly specialized regions at the plasma membrane that couple the intermediate filament cytoskeleton at points of strong cell–cell adhesion. Here, we review how desmosomes conferred new mechanical and signaling properties to vertebrate cells and tissues through their interactions with the existing junctional and cytoskeletal network.

## Introduction: overview of intercellular junctions

**Figure 1.**
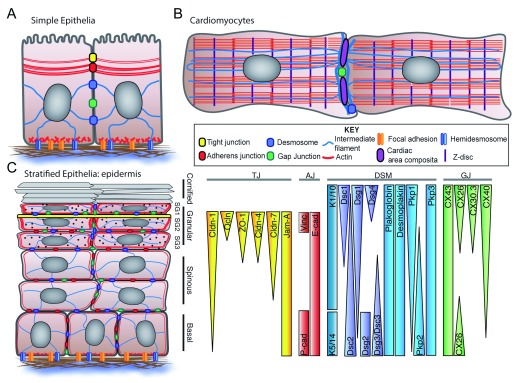
Organization of cell–cell junctions in different cell types. **A**) In simple epithelia, the junctional complex comprises apical tight junctions followed by adherens junctions and their attached cortical actin ring, and then desmosomes, which anchor the intermediate filament network. Gap junctions on the lateral membrane mediate the transfer of small molecules from cell to cell. Cell-matrix adhesion is facilitated by hemidesmosomes and integrin-based focal adhesions. **B**) In cardiac myocytes, contractile units called sarcomeres comprising thick (myosin) and thin (actin) filaments are joined at Z-discs and stabilized by interwoven desmin intermediate filaments. Actin fibers and intermediate filaments are anchored at hybrid junctions called *area composita* containing desmosome and adherens junction components. Stand-alone desmosomes anchor the remaining desmin-intermediate filament, and connexin (Cx)-containing gap junctions facilitate synchronous beating in heart tissue. **C**) Schematic of the epidermis and its multiple layers including the basal proliferating layer and differentiating spinous layer, granular layer, and fully differentiated cornified layer. Junctional proteins are polarized across multiple layers, as reported in 9– 12. The patterns of junctional proteins and their attached cytoskeletons help drive the differentiation process. cad, cadherin; Cldn, claudin; CX, connexin; Dsc, desmocollin; Dsg, desmoglein; Jam-A, junctional adhesion molecule A; K, keratin; Ocln, occludin; Pkp, plakophilin; SG, stratum granulosum; Vinc, vinculin; ZO-1, zonula occludens 1.

**Figure 2.**
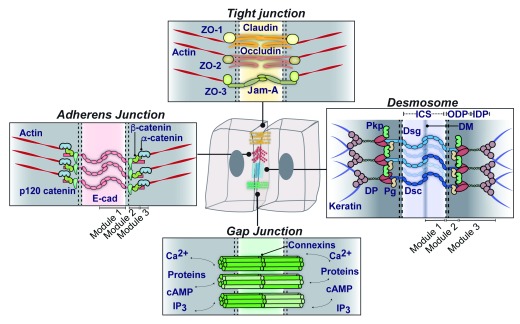
Schematics representing each of the major cell–cell junctions in simple epithelia and their major molecular components. Adherens junctions anchor the actin cytoskeleton at points of cell–cell adhesion, mediated by homophilic interactions between the classical cadherins (Module 1). The armadillo protein β-catenin (Module 2) and actin-binding protein α-catenin (Module 3) connect cadherins to the actin cytoskeleton. Similarly, in desmosomes, adhesion is mediated through desmosomal cadherins, desmogleins (Dsg) and desmocollins (Dsc) (Module 1), which associate with armadillo proteins plakoglobin (Pg) and plakophilins (Pkp) (Module 2) and desmoplakin (DP) (Module 3) to anchor keratin-containing intermediate filaments to the membrane. In tight junctions, claudins and occludin are tetraspan membrane proteins that help form the paracellular barrier, and junctional adhesion molecules (JAM-A) assist in their assembly. Zonula occludens (ZO) proteins fortify the junction from the cytosolic side. Gap junctions are built from homo- or hetero-hexamers of connexin proteins to form a dodecamer connexin channel complex. These channels allow the flow of ions, small molecules, and proteins between cells. cAMP, cyclic adenosine monophosphate; cad, cadherin; DM, dense midline; ICS, intercellular space; IDP, inner dense plaque; IP _3_, inositol trisphosphate; ODP, outer dense plaque.

Epithelia are essential for creating complex organs and organizing them into discrete compartments that allow functional diversification in metazoans. The ability of epithelia to perform these roles requires four types of macromolecular assemblies or intercellular junctions: desmosomes (DSMs), adherens junctions (AJs), tight junctions (TJs), and gap junctions (GJs) ^1– 6^. AJs and DSMs are anchoring junctions, which link the actin and intermediate filament (IF) cytoskeletons, respectively, to the plasma membrane at sites of cell–cell adhesion. TJs create seals in the plasma membrane to regulate paracellular transport and to polarize cells by keeping proteins in their correct compartments. Connexin-containing GJs electrically couple cells by allowing the movement of small molecules from cell to cell ( Figure 1 and Figure 2).

In simple epithelia, three intercellular junctions are organized into a polarized “junctional complex” at the apical end of the lateral membrane connecting two cells. TJs are most apical, followed by AJs and their associated actin belt, and finally DSMs ^7^. Spot DSMs and GJs are also present on other regions of the lateral membrane. In stratified epithelia, junctions are also organized in a polarized fashion but in this case across multiple cell layers, from the basal proliferating layer to the most apical differentiated layer ^8^ ( Figure 1). In cardiac myocytes, DSMs, AJs, and GJs are organized into a specialized region of the plasma membrane called the intercalated disc (ID), which synchronizes cardiac myocyte contraction by coupling mechanical and electrical functions ^13^ ( Figure 1). Based on the importance of intercellular junctions in the development of embryonic layers and in adult tissues, it is not surprising that interference with their structure and function contributes to many human diseases due to loss of tissue integrity or polarized cell functions ^3, 14, 15^.

**Figure 3.**
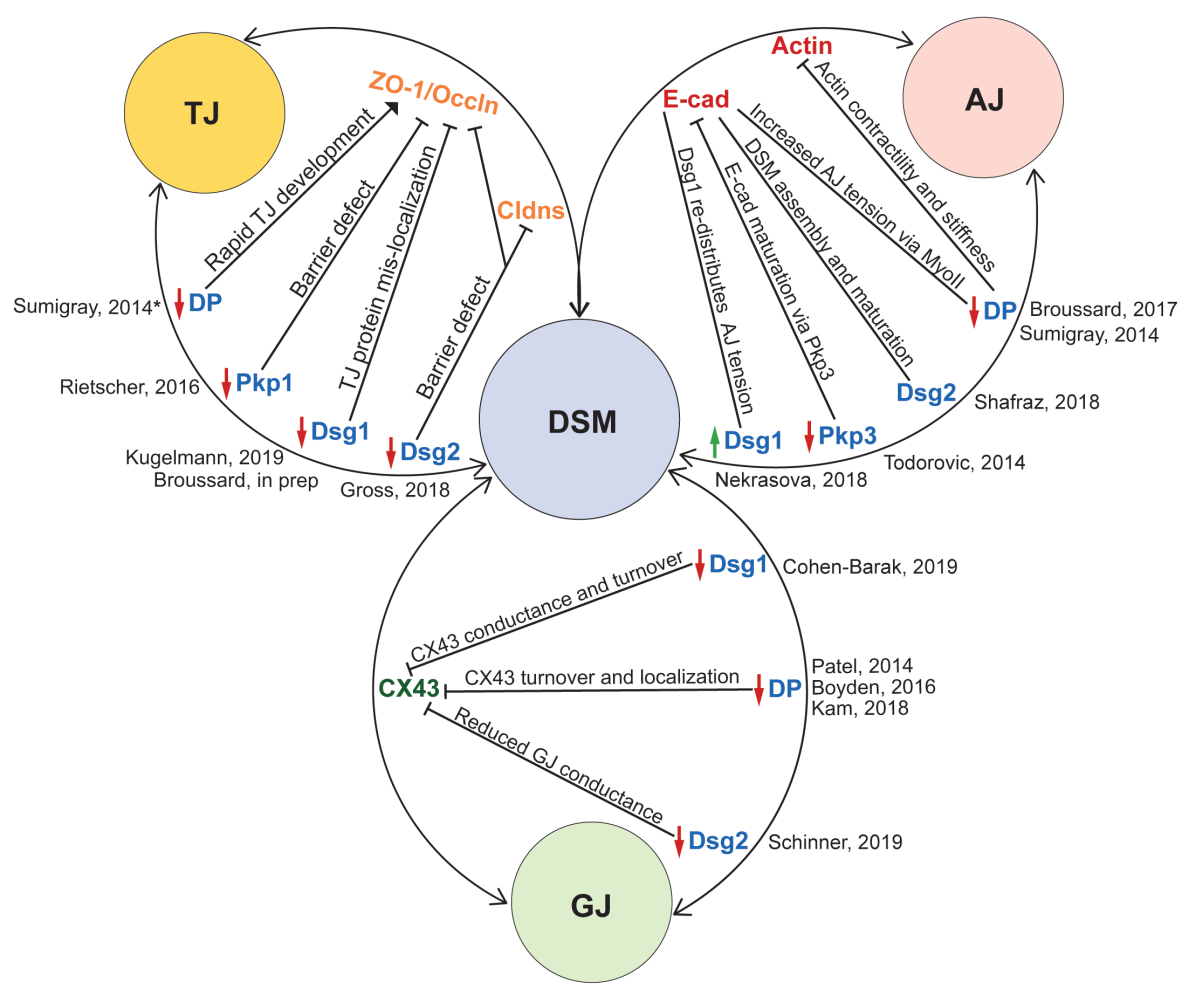
Structural and functional interactions between cell–cell junctions. Different components of cell–cell junctions work together to perform cellular processes and coordinate signaling events. From the perspective of the desmosome (DSM) (blue), here we list structural and/or functional relationships with tight junction (TJ) (yellow), adherens junction (AJ) (red), and gap junction (GJ) (green) components. Red and green arrows represent inhibition or activation, respectively, of DSM components. Black lines connecting DSM proteins with other junction components illustrate associations that promote or perturb junction form and function. The functional outcomes of these interactions are described over the connectors. Interactions represented in this figure are limited to those reported from 2014–2019. *Note: Sumigray *et al*. also reported a marked overexpression of claudins in desmoplakin (DP)-null keratinocytes. cad, cadherin; Cldns, claudins; CX43, connexin 43; Dsg, desmoglein; Occln, occludin; Pkp, plakophilin; ZO-1, zonula occludens 1.

In an historical overview of cell–cell junctions, Werner Franke and colleagues made an essential point: “It was the cooperation of the molecular ensembles of these junctions that provided the basis of eumetazoan life” ^16^. While it is widely recognized that junctions are not discrete, independently assembling and functioning structures, most reviews focus on junctions as distinct entities. Here we explore how DSMs, the evolutionarily most recent intercellular junctions, connect with AJs, TJs, and GJs to create a robust, functionally diverse system ( Figure 3).

## Desmosomes emerged from adherens junctions with structural similarities but distinct functions

Cadherins and armadillo proteins are ancient protein families that evolved independently but eventually came together to form primitive actin-associated AJs in simple epithelia of organisms such as Hydra and sponges ^17– 20^. Later, in vertebrates, DSMs used updated versions of cadherins and armadillo proteins, borrowed an IF-binding domain from the spectraplakin family, and put together a new junction important for integrity and differentiation in complex tissues ^20, 21^. DSMs share a modular structure with their relatives, the AJs ( Figure 2). At their adhesive core, both junctions have transmembrane components in the calcium-binding cadherin family, classic cadherins in AJs, and desmosomal cadherins in DSMs (Module 1), which associate in trans with cadherins on the adjacent cell to mediate adhesion. The cadherin cytoplasmic domain links to members of the armadillo family (Module 2), which together act as a platform for association with cytoskeletal adaptors (Module 3). These adapters associate with actin (i.e. α-catenin) in AJs or IF (i.e. desmoplakin) in DSMs ^7^.

### Several features set desmosomes apart from adherens junctions

First, their association with IF sets DSMs apart structurally and functionally from actin-associated intercellular junctions: IFs stretch to multiple times their original length without breaking and resist force at higher tensile loads than actin. Thus, the DSM–IF system imparts mechanical integrity to tissues (reviewed in Broussard *et al*., unpublished). These mechanical functions are critical in tissues that experience mechanical stress, such as the skin and heart. Second, there are two subclasses of desmosomal cadherins, desmogleins (Dsgs) and desmocollins (Dscs), both of which are required for DSM structure and function. Third, DSMs are uniquely capable of undergoing a transition from calcium-dependent to calcium-independent status, also known as the “hyperadhesive” state ^1, 22^. Fourth, DSMs, but not AJs, segregate into lipid raft regions of the plasma membrane ^23^. The resulting intercellular junctions are highly ordered, bilaterally symmetrical plasma membrane-associated organelles that anchor 10 nm IFs to multi-layered electron-dense cytoplasmic plaques ^24^. The following sections introduce the protein families that make up DSMs.

### The cadherins

A major breakthrough in our understanding of DSM diversity occurred when it became apparent that multiple members of the Dsg and Dsc subclasses of cadherins exist, which are expressed in a stratification-specific distribution in the multi-layered epidermis ^4, 25, 26^. The pattern has biological meaning tied to the differentiation program itself ^27^ ( Figure 1). The pattern also has functional importance in disease. For instance, the layer-specific nature of skin lesions in the autoimmune diseases pemphigus vulgaris and pemphigus foliaceus correspond to the layers in which the target antigen (i.e. Dsg3 versus Dsg1) is at its highest concentration ^28, 29^.

Desmosomal cadherins evolved features not present in classic cadherins, tailoring their functions in vertebrates. Structural studies suggest that Dsgs and Dscs interact in trans via a strand swap mechanism, mimicking the interaction mechanism of classic cadherins. However, a pattern of conserved charged amino acids in their ectodomains inhibits homophilic interactions such that heterophilic interactions are preferred (though evidence for the latter also exists) ^30^. Other studies suggested that the Dsg2 ectodomain is flexible even in the calcium-bound state and shorter than the ectodomains of classic cadherins ^31^. The data are consistent with the idea that desmosomal cadherins form trans but not cis interactions because they are spaced too far apart. Desmosomal cadherin flexibility may contribute to a switch between adhesive states during processes such as wound healing.

The cytoplasmic domains of the desmosomal cadherins are also different, with an extended Dsg-unique tail that scaffolds a number of binding partners involved in growth factor signaling and cytoskeletal remodeling (reviewed in 32). The Dsc tail size is similar to that of classic cadherins but has two splice isoforms, whose functions are poorly understood. Dsc2, however, is sufficient to recruit and organize complexes containing the IF-anchoring protein desmoplakin (DP) on patterned substrates ^33^.

### The armadillo proteins

Like AJs, DSMs have two sorts of armadillo proteins with junctional and nuclear functions (reviewed in 34, 35) ( Figure 2). Plakoglobin and β-catenin are paralogues, and the plakophilins (PKPs) are most comparable to p120 catenin, owing to their similar structure of central armadillo repeats. Both plakoglobin and the PKPs associate directly with the IF-anchoring protein DP and interact with each other. Together, these interactions complete a link between the plasma membrane and IFs and promote clustering of plaque proteins in the cell cortex. Whereas β-catenin typically associates only with classic cadherins, plakoglobin is capable of associating with both desmosomal and classic cadherins ^36^. This feature may be important in coordinating the assembly of AJs and DSMs.

PKPs contribute to junctional diversity in the epidermis and heart. In the epidermis, PKP1–3 are expressed in a differentiation-dependent manner ( Figure 1). PKP2 is expressed at low levels in the epidermal basal layer, and patients harboring PKP2 mutations have cardiac symptoms without visible alterations in the epidermis, likely due to the compensatory presence of other PKPs ^34^. Human and animal studies show that PKP1 plays a critical role in ectodermal structural integrity, barrier function, and growth control ^37^. PKP1 also exhibits differentiation-dependent localization in the epidermis: nuclear in basal cells and junctional in suprabasal cells. The functional importance of this transition is poorly understood. Global PKP3 knockout mice are viable, but their skin exhibits features of human atopic dermatitis ^38^. While p120 catenin and PKPs both regulate Rho GTPases, many of their functions are different, and we are only just beginning to appreciate the additional diversity these plaque elements bring to the structural and signaling functions of DSMs ^34^.

In cardiac myocytes, a mixed type of junction, the area composita, brings together DSM (DP, PKP2, Dsg2/Dsc2/plakoglobin) and AJ (N-cadherin, β-, and αT-catenin) components through PKP2’s ability to associate with αT-catenin in cardiac cells ^39^. The intermingling of AJ and DSM components results in the attachment of desmin IFs and actin microfilaments within the same plasma membrane domain, providing additional support for actively beating cardiac myocytes. The identification of the area composita occurred in parallel with the recognition of a cardiac disorder called arrhythmogenic cardiomyopathy (AC), associated with mutations in DSM molecules. With these events came an explosion of studies focused on the specialized junctions of the heart and DSM mutations in AC ^40^.

### The cytoskeletal adaptor protein

DP is an essential mediator of IF anchorage at DSMs. Its domain structure evolved from the more primitive spectraplakin family, which can be found in non-vertebrates such as *Caenorhabditis elegans* ^41^. The IF-binding domain exhibits broad functionality, binding to IFs in most classes ^42^. DP associates with all of the armadillo proteins directly through its amino terminus, associations that are regulated by post-translational modifications ^43^. The spectrin repeats in the DP N-terminus also associate with binding partners such as the microtubule plus-tip protein EB1, which stabilizes microtubules to promote Cx43 trafficking to the membrane (see below) ^44^.

## Desmosomes and adherens junctions: integration of junction dynamics

DSM dynamics (assembly and turnover) have been studied mostly *in vitro*, using calcium levels, growth factor stimulation or inhibition, and scrape wounding, as a means to manipulate junction status ^26^. In general, the formation of DSMs is considered to be dependent on classic cadherin-mediated adhesion, since cells lacking classic cadherins do not assemble DSMs ^45^. Live-cell imaging at the newly contacting edge of a scrape wound shows fluorescently tagged E-cadherin arriving at cell–cell interfaces within 3 minutes, along with associated β-catenin, and perhaps plakoglobin. Indeed, it has been proposed that DSM and AJ assembly crosstalk depends on the armadillo protein plakoglobin ^46^, which has the special ability to associate with both classic and desmosomal cadherins. At the same time, PKP2 accumulates at nascent borders in concert with RhoA and the coalescence of circumferential actin bundles under the membrane ^47^. These PKP2-dependent actin rearrangements help transport hundreds of small macromolecular particles containing DP and PKP2 associated with tufts of IFs towards the forming cell–cell adhesion to reinforce the desmosomal plaque ^48^.

In PKP3-deficient cells, the ordered recruitment of DP into small precursor particles does not occur. Instead, DP coalescences aberrantly at cell–cell borders, which is reversed by activating cAMP with forskolin. Rather than utilizing the PKA pathway, cAMP signaling engages EPAC and its downstream effector, the small GTPase Rap ^49^. While most literature focuses on how DSMs hitchhike on AJ assembly, here, data support the idea that, in addition to regulating DP precursor assembly, PKP3 provides a scaffold for binding and activation of Rap1 required for E-cadherin association, which in turn is necessary for concentrating E-cadherin at AJs. PKP3 also creates more dynamic DSMs compared with PKP1, which promotes robust adhesion ^50^. Growth factor-dependent phosphorylation of PKP1 and PKP3 dictates their association with different 14-3-3 isoforms to promote either PKP1’s disassociation from DSMs or PKP3’s association with a special site at the nexus of three cells called tricellular contact sites ^51^. These associations either dial down or dial up DSM stability and adhesion.

The availability of assembly-competent DP is also determined by post-translational modifications within a signaling hub in the DP C-terminus. Phosphorylation of DP S2849 cooperates with upstream arginine methylation to drive the phosphorylation of an intervening cascade of serines by GSK3β. The fully phosphorylated state is more dynamically associated with IFs and assembles more efficiently into DSMs ^52^. While the phospho-deficient DP mutant S2849G incorporates more slowly into DSMs owing to its accumulation on cytoplasmic IFs, once incorporated, it mediates a stronger association with IFs and strengthens adhesion ^53^. Interestingly, one of the key arginine methylation sites is a target for mutation in AC, underscoring the critical nature of this regulatory mechanism *in vivo* ^54^.

Dsgs incorporate into DSMs independently from the DP/PKP2 complex in response to cell–cell contact ^48, 55^. Whereas DP/PKP2 depend on actin for their assembly into junctions, the desmosomal cadherins require microtubules and, in the case of Dsg2/Dsc2, move outwardly on kinesins to the plasma membrane. At least some Dsgs (Dsg2) can associate with E-cadherin, and this interaction may help initiate early stages of DSM assembly ^56^. How these events are coordinated temporally and spatially, particularly in complex stratified tissues, is poorly understood.

While interactions between Dsg2 and E-cadherin may be a transient step in the assembly of DSMs, desmosomal components constitutively associate with other types of cadherin-containing junctions. In addition to its association with N-cadherin/catenin complexes in the area composita, as discussed above, DP associates with VE-cadherin via plakoglobin in certain endothelial cells to strengthen endothelial adhesions through association with vimentin IFs ^57^. Furthermore, PKP2 is in several types of N-cadherin-based junctions in the retina, astrocytes, and mesenchymal cells ^58, 59^. Finally, force-associated attachment of keratins with C-cadherin in *Xenopus*, through plakoglobin, is important for the formation of polarized protrusions and persistent migration within a collectively migrating tissue ^60^.

Just as classic cadherins and associated cortical actin contribute to DSM assembly, actin is also involved in DSM de-stabilization. Reduced calcium, tyrosine kinases, including growth factor receptors such as EGFR, proteases (MMPs, ADAMs, and bacterial toxins), autoimmune antibodies, and regulators of endocytosis have all been implicated in DSM turnover, frequently also involving the actin cytoskeleton associated with AJs ^4, 61, 62^.

For instance, loss of DSM adhesion via pemphigus vulgaris autoantibodies targeting Dsg3 involves impairment of the actin cytoskeleton and is restored by activating RhoA ^63^. Furthermore, serine phosphorylation of the actin-associated protein adducin due to calcium influx and PKC activity is protective in pemphigus ^64^. This positive role for PKC in adhesion is interesting in light of its known role in increasing DSM dynamics and calcium chelation-dependent internalization of half DSMs ^61^. PKC-sensitized internalization of DSMs is thought to occur *in vivo* during wound healing and in response to loss of keratins or transition to wound-healing keratins ^65, 66^.

Differential downregulation of E-cadherin and Dsgs by EGF was recently reported to result in entry of Dsg2 into a recycling pool, while E-cadherin is cleaved by MMPs and degraded ^67^. In this case, initial disappearance of Dsg2 was observed *in vitro* and *in vivo* at the leading edge of the wound. Dsgs also destabilize through loss of the primary cilia component RPGRIP1L, which normally inhibits Dsg3 turnover by blocking endocytosis ^68^.

In the gut, the enteropathogenic *Escherichia coli* (EPEC) bacterium interferes with DSM stability by inhibiting Rho GTPases through the effector protein EspH ^69^. On the other hand, complete loss of DP from gut epithelium did not lead to overt adhesion defects. However, microvilli were significantly shorter in these animals, suggesting crosstalk with actin-based structures with a potential impact on gut homeostasis ^70^.

In our next section, we discuss how DSMs and DSM–IF interactions coordinate with AJs and actin to alter cell and tissue behavior.

## Coordination of desmosome and adherens junction functions in cytoskeletal control of cell behavior and signaling

IFs are networked through direct and indirect interactions with other non-muscle cytoskeletal elements, actin and microtubules, and their assembly and behavior depend on these interconnections ^71^. In particular, association of the DSM–IF complex with actin-linked adhesions is bolstered upon application of physical force ^60, 72^. In turn, the DSM–IF network can modulate the mechanical properties of cells but may require actin to do so.

For instance, mutations in the IF-anchoring protein DP that either dial up (DP S2849G mimicking a hypophosphorylated form of DP that binds more robustly to IFs) or dial down (DP-NTP which uncouples IFs from the DSM) DP–IF interactions result in increased or decreased stiffness and cell–cell adhesion forces, respectively ^73^. PKCα is involved in mediating a switch between hyperadhesive and dynamic DSMs by modulating DP–IF interactions: PKC inhibition increases adhesive strength to promote hyperadhesion, whereas PKC activation stimulates DSM dynamics ^1, 74^. Thus, PKCα can act as a rheostat to influence biological outcomes. Alterations in cell mechanical properties downstream of the DP–IF interaction depend on an intact actin cytoskeleton ^73^. Since it has been suggested that the IF system can resist forces generated by the actin system ^75^, these data are consistent with the idea that the contractile actin cytoskeleton works against the DSM–IF network to tune cell mechanics.

What actin regulators are involved in integrating AJs and DSMs? AJs and TJs are well established to recruit Rho GTPase regulators such as RhoGEFs and GAPs important for junction assembly and regulation of intracellular signaling ^76^. However, DSMs also interact with RhoA signaling mediators. As mentioned above, inhibition of RhoA via an enteropathogen destabilizes DSMs in the gut. Furthermore, desmosomal proteins such as PKP2 regulate small GTPases in the Rho family to modulate actin-dependent assembly of the desmosomal plaque ^47^. PKP2 also brings PKC to DP to regulate its dynamics and junction stability; thus, PKP2 may functionally link these two pathways ^74^. A potential RhoA regulator at DSMs is the GEF Ect2. Ect2 was previously shown to be recruited by α-catenin to the AJ, where the complex regulates membrane tension ^77^. Ect2 also associates with DP in cardiac muscle to control actin polymerization and in keratinocytes where it controls adhesion (Kam, Zarkoob, *et al*., unpublished data). The extent to which classic and desmosomal cadherin-dependent recruitment of RhoGEFs and GAPs are integrated awaits further study.

The importance of desmosomal control of actin remodeling is apparent in the physiologic context of epidermal differentiation. In basal cells, decreased cortical tension coupled with increased classic cadherin adhesion drive differentiation and delamination to promote stratification ^78^. In addition, however, overlaid onto the classic cadherins are multiple desmosomal cadherins that are expressed in tissue-specific and differentiation-dependent patterns. This desmosomal cadherin patterning also helps drive differentiation and morphogenesis ^9^ ( Figure 1). For instance, Dsg1, which is present only in stratified tissues of mammals, is first expressed as basal cells of the epidermis commit to differentiate and stratify. The onset of Dsg1 expression during epidermal differentiation corresponds with re-organization of the actin cytoskeleton via Arp2/3-dependent actin polymerization near DSMs. This new actin polymerization near DSMs is associated with a reduction of tension on AJs as measured by an E-cadherin FRET sensor and reduced vinculin staining. The biological consequence of these actin rearrangements is delamination of basal cells into superficial layers of the epidermis ^79^. The question remains, however, whether the DP–IF connection is required for this process. Consistent with its importance in morphogenesis, DP was recently shown to drive the process of radial intercalation where basally located cells move into the outer epidermal layer in *Xenopus* ^80^.

In cardiac muscle cells, DP associates with Myozap (myocardium-enriched zonula occludens-1 [ZO-1]-interacting protein), which in turn associates with Dysbindin. Dysbindin interacts with RhoA to affect SRF-dependent transcription and induce cardiac hypertrophy ^81^. Other signaling pathways contributing to AC have been attributed to DSM dysfunction. This includes the Hippo pathway, which is activated in PKP2-deficient cardiac myocytes and which contributes to adipogenesis in AC ^82^. Another major source of signaling regulation important for cutaneous and heart biology is the β-catenin/WNT pathway, which is impacted by changes in the DSM molecule plakoglobin. For details of this large subject area, we refer the reader to recent reviews ^83, 84^.

Finally, DSM molecules also regulate actomyosin signaling and function in single cells, suggesting that they work together with not only cell–cell AJs but also the cytoskeleton associated with focal contacts and ECM. This was shown previously for plakoglobin ^85^, and more recently for DP, which through its ability to regulate RhoA and p38 MAPK signaling controls epithelial cell migration ^86^.

## Desmosomes and connexins/gap junctions

The importance of E-cadherin for promoting connexin (Cx) dynamics and GJ assembly was recognized first in the early 90’s ^87, 88^. Recently, a provocative report showed that Cxs also regulate cadherins: a nuclear-localized piece of the Cx43 C-terminus forms a complex with basic transcription factor-3 and PolII to regulate N-cadherin gene transcription ^89^.

DSMs also contribute to Cx assembly and GJ function. Interfering with this relationship through mutation of DSM components is an important contributor to skin and heart disease ^13, 90, 91^. A connection between the DSM and Cxs was first made in the heart, showing that PKP2 is critical for Cx43 expression and function ^92, 93^. High-resolution STORM microscopy revealed an intimate physical association between PKP2 and Cx43 in molecular clusters scaffolded by Ankyrin-G and containing the sodium channel NaV1.5 ^94^. These complexes can occur outside of GJ plaques in what has been referred to as the “connexome” ^93^. These data are consistent with the observation that PKP2 regulates the sodium current and action potential velocity. Thus, DSM molecules help integrate mechanical and chemical signals at the ID ^95, 96^.

Desmosomal cadherins, DP, and plakoglobin also functionally link to Cx43 and GJs. Dsg2 co-immunoprecipitates with Cx43, and knockdown of Dsg2 or its associated protein plakoglobin interfered with the rhythmic beating and reduced the velocity of excitation propagation in HL-1 cardiac myocytes. This effect was rescued through adrenergic signaling stimulated by cAMP elevation ^97^. Dsg2 and NaV1.5 also co-immunoprecipitate, and Dsg2 mutant mice exhibited reduced action potential due to a lower sodium current density ^98^.

DP regulates Cxs in the heart and skin through multiple mechanisms. It interferes with Cx43 turnover by attenuating a Ras/pErk pathway that leads to Cx43 phosphorylation, priming it for internalization ^99^. In the epidermis, DP also regulates Cx43 by dampening pErk, but in this case something other than Ras seems to be the upstream stimulus for Erk activation in DP-deficient keratinocytes. These results raise the possibility that DSM control over junctions exhibits both shared and tissue-specific components ^99^. DP also promotes Cx43 delivery to the plasma membrane through its ability to stabilize junctional microtubules by binding the microtubule plus-tip protein EB1 ^44^. Interfering with DP binding to EB1 through point mutations in the DP N-terminus does not affect DP localization at borders but results in unstable microtubules and loss of border-localized Cx43 and GJ coupling ^44^. As DP loss also reduces NaV1.5 on the plasma membrane in HL-1 cells ^100^, it would be interesting if microtubule instability underlies NaV1.5 reduction.

DP and Dsg1 mutations have recently been shown to result in systemic disorders such as severe dermatitis, multiple allergies, and metabolic wasting (SAM) syndrome and erythrokeratodermia-cardiomyopathy syndrome, some of which are also associated with perturbation of Cx43 and GJ function ^101– 104^. In fact, some SAM patients exhibit epidermal skin lesions reminiscent of erythrokeratodermia variabilis caused by Cx mutations ^105^. In these cases, Dsg1 reduction impaired Cx43 expression and localization, possibly through PKC-dependent phosphorylation and de-stabilization of Cx43. Importantly, there were no observed changes in other DSM molecules, and loss of Dsg1 did not reduce adhesion in an *in vitro* test of cell–cell adhesion, so the results are unlikely to be due to a generalized loss of adhesion.

In the future, it will be important to determine the extent to which these connections reflect direct interactions between DSM and components outside of their normal junctional structures, as proposed for the “connexome” in cardiac myocytes.

## Desmosomes and tight junctions

TJs are made of an interconnected network of protein strands that on cross section appear as “kissing points” that seal adjoining plasma membranes and create selective ion permeability barriers between epithelial or endothelial cells ^7, 106^. It has been known for some time that TJs form in simple polarized epithelial cells in parallel with the formation of the AJ-dependent actin re-organization (reviewed in 6). Actomyosin contraction not only regulates cadherin-based junction dynamics but also is critical for TJ function and responses to external stimuli ^107^.

The dependence of TJs on AJs could be due to a number of factors. These include AJ-mediated recruitment of signaling molecules (e.g. RhoGTPases, GAPs, and GEFs), assembly of Par polarity protein complexes necessary for TJ positioning, microtubule rearrangements that initiate trafficking of TJ membrane proteins like claudins (although the TJ protein cingulin can recruit microtubules on its own ^108^), or AJ-dependent alterations in lipid content (e.g. increased cholesterol content ^6^). The dependence of TJs on lipid content is consistent with the reported partitioning of TJs to lipid rafts. DSMs also preferentially partition into lipid rafts ^23, 109, 110^, but whether or not this or other aspects of DSM biology impact TJ assembly is only now being addressed.

As described above, DSMs have been shown to control microtubule stability through EB1 binding to DP, which affects Cx trafficking, but could also promote trafficking of TJ proteins ^44^. DP also stimulates cortical microtubule assembly *in vivo* in mouse epidermis by recruitment of centrosomal proteins such as Lis1 to the plasma membrane as cells differentiate ^111– 114^. This DP-dependent rearrangement of microtubules in turn recruits myosin II to junctions, which causes tension-induced AJ strengthening associated with elevated TJ function as measured by TEER ^113^. DP–IF attachments also engage AJ and cortical actin, promoting cell stiffening and increased force on cell–cell junctions. These outcomes depend on the presence of a functional actin contractile system ^73^. As DP–keratin interactions become more robust with differentiation, it is possible that actin uses this evolving DP–IF system to increase tension in the system, which in turn helps to increase TJ integrity and possibly restrict TJ assembly to the granular layers, as discussed below.

In the epidermis, continuous TJ strands are restricted to one of three superficial keratinocyte layers in the multicellular epithelium, the stratum granulosum 2 (SG2) layer ^115^. This special layer separates an aqueous environment below and the physical stratum corneum barrier above. The restricted positioning of TJs to this layer likely depends on a number of factors. For instance, E-cadherin integrates EGFR and actin-based mechanical signaling to inhibit TJ formation in lower layers while increasing tension and TJ stability in the SG2 layer ^116^. Interfering with the superficial DSM cadherin Dsg1 has a similar impact on TJs: in both cases, the TJ protein ZO-1 accumulates aberrantly in the spinous layer, losing its restricted positioning in the SG2 layer (Broussard *et al*., unpublished data). The importance of Dsg1 for TJs was also observed in Dsg1 knockout animal models ^117^ (Godsel, Roth-Carter *et al*., unpublished data). Loss of PKP1, which associates with both DP and Dsg1, also results in impaired TJ function ^118^. Whether it is the role of PKP1 in enhancing Dsg1–DP clustering activity ^119^ or some other independent function, or both, awaits further study.

Functional interactions between DSMs and TJs also exist in another regenerating tissue, the gut. Dsg2/Dsc2 expression is reduced in the intestinal epithelia of patients with the inflammatory condition Crohn’s disease ^120, 121^. Tissue-specific deletion of Dsg2 and Dsc2 in mice showed that Dsg2 but not Dsc2 is required for TJ barrier development/maintenance ^121^. Dsg2’s regulation of the barrier was attributed to its ability to control EGFR activation through Src ^120^, and more recently to control p38 MAPK ^122^. Another recent report showed that KLF5 mediates the transcription of Dsg2, which in turn promotes the TJ barrier; barrier loss due to KLF5 ablation could be partially restored by re-introducing Dsg2 ^123^. Thus, desmosomal molecules and their regulatory pathways are potential new targets for developing treatments for patients with barrier-disrupting intestinal disorders.

In the heart, proteins that make up epithelial TJs are present in the ID and act as connectors and scaffolds for other intercellular junction components, but do not form TJs ^15^. These include ZO-1, which is a TJ plaque protein but is also associated with AJ proteins and Cxs. In cardiac IDs, ZO-1 is linked to the DSM protein DP by Myozap ^124^. Myozap deficiency results in severe contractile dysfunction in zebrafish ^124^, whereas overexpression of Myozap in mice results in protein-aggregate-associated cardiomyopathy ^125^. This example highlights the interconnectedness of proteins at the ID, where intercellular junction proteins reach the height of structural and functional interactions.

## Interfering with the integrated DSM-junction network in human disease: more than just adhesion?

Based on the work discussed in preceding sections, it is clear that the integrated DSM network is a target in skin, heart, and gut disorders. While many biological outcomes have been attributed to loss of cell–cell adhesion in general, molecule-specific functions that supersede adhesion and impinge on multiple junction types are emerging ^40, 126– 128^. These functions include regulation of gene transcription, protein translation, cell growth, differentiation, apoptosis, motility and invasion, and cancer metastasis ^32, 118, 129– 132^.

In the autoimmune skin disease pemphigus, pathogenic autoantibodies result in blistering, likely through a combination of interference with adhesion, targeted turnover of Dsgs, and signaling pathways that are elicited downstream of antibody binding. Several target pathways have been identified, such as calcium p38MAPK, RhoA, PKC, Src, EGFR/Erk, and p53 (reviewed in 62 and see below 133). Most of these signaling mediators have been linked not only to DSMs in other contexts such as epidermal differentiation and wound healing but also to other intercellular junctions through either genetic manipulation or disease (reviewed in 6, 15, 134).

Similarly, in AC, many normal developmental signaling pathways are altered in response to DSM mutations, and these can have broad consequences for cardiac myocyte functioning and cell fate by connecting the ID with mechanical signaling ^135, 136^. For instance, cardiac myocyte identity depends on coupling the ID with RhoA‐ROCK/actin to govern gene transcription via the transcription factor MRTF/SRF ^136^. PKP2 loss in cardiac myocytes also elevates TGFbeta and p38MAPK signaling, which together coordinate a transcriptional program resulting in pro-fibrotic gene expression. Importantly, DP levels were reduced under these conditions, and re-introduction of DP restored normal levels of this signaling cascade ^137^. PKP2 also controls the transcription of genes involved in calcium cycling and cardiac rhythm ^138, 139^. As mentioned above, PKP2 mutations in AC have also been linked to alterations in Hippo/Wnt signaling ^82^, and GSK3 inhibitors can reverse AC phenotypes caused by plakoglobin and DP mutations ^82^. In some cases, defects associated with impairment of the interconnected junction network can be reversed by specific treatments. For instance, in cardiac muscle cells, activating adrenergic receptors in plakoglobin/Dsg2-depleted cells restores beating ^97^. These studies not only expand our understanding of DSM function but also reveal potential targets for new therapeutic approaches to ameliorate disease.

## Prospects and questions for the future

We have learned a lot about how intercellular junctions are integrated to support normal tissue morphogenesis and homeostasis. However, there are many questions remaining. Just as the DSM–IF system interacts with actin and microtubules, the DSM and its associated intercellular junction network interact with cell–substrate adhesions, including focal contacts and hemidesmosomes. Cell–cell and cell–substrate forces must be balanced in biological systems, and thus there is much to be done in the future to determine how the entire junctional network is integrated.

The extent to which DSMs transduce mechanical signals directly, or simply modulate mechanical signals through their interactions with other cytoskeletal elements, is an area just now being addressed using newly developed desmosomal cadherin and DP tension sensors (reviewed in Broussard *et al*., unpublished; ^140, 141^). While a tension sensor module placed just upstream of the IF-binding domain indicates that DP becomes mechanically loaded only when cells experience external stress, molecular dynamics simulations suggest that the N-terminal SH3 domain could act as a force sensor and perform a stabilizing role ^142^. Whether this region of DP behaves as a mechanosensor awaits experimental tests.

Another fertile area for future study is to address how DSMs function not only to resist stress but also to sense and respond to stress. A role for DSMs as stress sensors is consistent with their appearance in evolution at a time when there was an expansion of epithelial tissue complexity, UV irradiation from sunlight, and new microbes. Evolution of the circulatory system and the formation of hearts with multiple chambers as well as the development of a more complex immune system also occurred during this time (reviewed in 20).

Two examples supporting the idea of DSMs as stress sensors and responders come from studies focused on Dsg3 and Dsg1. These desmosomal cadherins are found specifically in complex stratified epithelia such as the oral mucosa and skin, two tissues that experience multiple types of stress stimuli. Dsg3, which is concentrated in basal proliferating keratinocytes, was recently shown to protect cells from stress including that caused by UV irradiation ^133^. It does so by keeping the reins on p53; knockdown of Dsg3 *in vitro* or its ablation *in vivo* resulted in elevated p53, reduction in cell cycle regulators, and increased indicators of apoptosis. Elevated p53 also occurred in response to treatment with pemphigus vulgaris antibodies, raising the possibility that Dsg3’s ability to regulate p53 is broadly applicable to multiple stress stimuli targeting this desmosomal cadherin ^133^.

While Dsg3 levels remain constant in response to UV stress ^133, 143^, expression of the desmosomal cadherin Dsg1 is reduced under these conditions. Modeling UV-mediated stress, genetic depletion of Dsg1 was shown to increase pro-inflammatory secreted factors, similar to those stimulated by UV. In addition, these factors signal in a paracrine fashion to melanocytes. Conditioned media from Dsg1-deficient keratinocytes stimulated melanocyte dendritogenesis and pigment secretion from normal melanocytes, both of which occur normally in response to UV to promote the transfer of pigment to neighboring keratinocytes ^144^. Thus, loss of keratinocyte Dsg1 in response to UV-mediated stress may serve as a sensor that elicits a protective response in other cell types within the tissue microenvironment.

Similarly, Dsg1 loss in SAM syndrome, a disorder caused by bi-allelic loss of Dsg1 or mutations in DP resulting in Dsg1 depletion, results in not only physical barrier defects but also increased pro-allergic and pro-inflammatory cytokines produced by keratinocytes in a cell-autonomous fashion ^101, 103^. These cytokines could have multiple biological outcomes, from decreasing TJ and Cx/GJ assembly and function to the recruitment of immune cells into the affected epidermis ^105, 128^. Indeed, targeting cytokine networks can ameliorate symptoms in patients with skin disease due to DP mutations ^145^.

DSM response to stress can be paracrine in nature in both the heart and the skin. For instance, PKP2 knockdown stimulated ATP release at least in part through Cx43 and resulted in overexpression of genes involved in adenosine-receptor cascades. This paracrine pathway contributes to fibrosis and impaired cardiac function in PKP2 knockout animals ^146^. These data suggest that stimulation of paracrine signals in response to DSM mutations in cardiocutaneous and cardiac disorders may cooperate with altered mechanical signaling to contribute to disease pathogenesis.

While we focused primarily on downstream events stimulated by junction interactions or modulation, upstream stress sensing also regulates intercellular junctions. For example, the transcription factor Nrf2, which controls genes involved in antioxidant defense, increases the expression of the microRNAs miR-29a and miR-29b in keratinocytes to target hyperadhesion by decreasing Dsc2 ^147^.

In conclusion, it is clear that intercellular junctions have evolved to meet the increasingly complex needs of the organism by developing new ways of communicating and cooperating with other junctions. It is also clear that we have only skimmed the surface of the complexity of cell junctional networks. Future work will be advanced by the availability of sophisticated new optical imaging probes and sensors, by BioID methods to characterize nearest neighbors in the junction network, and by state-of-the art methods for genetic interference and editing as well as single cell analysis of complex tissues.

## Abbreviations

AC, arrhythmogenic cardiomyopathy; AJ, adherens junction; Cx, connexin; DP, desmoplakin; Dsc, desmocollin; Dsg, desmoglein; DSM, desmosome; GJ, gap junction, ID, intercalated disc; IF, intermediate filament; PKP, plakophilin; SAM syndrome, severe dermatitis, multiple allergies, and metabolic wasting syndrome; SG, stratum granulosum; TJ, tight junction; ZO-1, zonula occludens-1.
